# Mobile phone text messaging for promoting adherence to anti-tuberculosis treatment: a systematic review protocol

**DOI:** 10.1186/2046-4053-2-6

**Published:** 2013-01-16

**Authors:** Mweete D Nglazi, Linda-Gail Bekker, Robin Wood, Gregory D Hussey, Charles S Wiysonge

**Affiliations:** 1 The Desmond Tutu HIV Centre, Institute for Infectious Disease and Molecular Medicine and the Department of Medicine, Faculty of Health Sciences, University of Cape Town, Anzio Road, Observatory, 7925, Cape Town, South Africa; 2 International Union against Tuberculosis and Lung Disease, 68 Boulevard Saint-Michel, 75006, Paris, France; 3 Vaccines for Africa Initiative, Institute of Infectious Disease and Molecular Medicine, Faculty of Health Sciences, University of Cape Town, Anzio Road, Observatory, 7925, Cape Town, South Africa; 4 Department of Clinical Laboratory Sciences, Division of Medical Microbiology, Faculty of Health Sciences, University of Cape Town, Anzio Road, Observatory, 7925, Cape Town, South Africa

**Keywords:** Mobile phone, Text messages, Tuberculosis treatment, Anti-tubercular agents, Adherence, Compliance

## Abstract

**Background:**

In 2010, there were approximately 8.8 million incident cases of tuberculosis (TB) worldwide. The treatment of TB is at least six months long and may be complicated by a high pill burden. In addition, TB patients often do not take their medication on schedule simply because they forget. Mobile phone text messaging has the potential to help promote TB treatment adherence. We, therefore, propose to conduct a review of current best evidence for the use of mobile phone text messaging to promote patient adherence to TB treatment.

**Methods:**

This is a systematic review of the literature. We will preferably include randomized controlled trials (RCTs). However, non-randomized studies (NRS) will be considered if there is an inadequate number of RCTs.

We will search PubMed, EMBASE, CINAHL, CENTRAL, Science Citation Index, Africa-Wide Information, and WHOLIS electronic databases for eligible studies available by 30 November 2012 regardless of language or publication status. We will also check reference lists for additional studies, identify abstracts from conference proceedings and communicate with authors for any relevant material.

At least two authors will independently screen search outputs, select studies, extract data and assess the risk of bias (using separate criteria for RCTs and NRS); resolving discrepancies by discussion and consensus. We will assess clinical heterogeneity by examining the types of participants, interventions and outcomes in each study and pool studies judged to be clinically homogenous. We will also assess statistical heterogeneity using the chi-square test of homogeneity and quantify it using the I-square statistic. If study results are found to be statistically homogeneous (that is heterogeneity *P* > 0.1), we will pool them using the fixed-effect meta-analysis. Otherwise, we will use random-effects meta-analysis. We will calculate risk ratios and their corresponding 95% confidence intervals for dichotomous outcomes, and mean differences for continuous outcomes. For other outcomes without quantitative data, a descriptive analysis will be used.

**Discussion:**

Our results can be used by researchers and policy-makers to help inform them of the efficacy of mobile phone text messaging interventions to promote patient adherence to TB treatment.

## Background

Tuberculosis (TB) is a major public health concern, with an estimated 8.8 million incident cases and 1.7 million deaths each year worldwide [1,2]. The burden of TB is highest in 22 low- and middle-income countries, mostly located in sub-Saharan Africa, where TB is fuelled by the HIV/AIDS epidemic [2].

The World Health Organization (WHO) guidelines for TB treatment recommend a directly observed treatment short course (DOTS) strategy to monitor patient adherence to medication [1,3]. This strategy includes treating TB using standardized rifampicin-based regimens of six months duration for new TB cases and eight months for retreatment cases. Failure of patients to complete TB treatment results in infectivity, drug resistance, relapse and death [4]. It is therefore important to find better ways of improving patient adherence to TB treatment.

A variety of factors may impact on patient adherence to medication, and thus efforts to improve medication adherence in general are more effective when they address multiple dimensions of adherence behaviours rather than single-target interventions [5,6]. Several strategies promoting TB medication adherence have been investigated. These include interventions promoting better health care provider-patient communication about adherence; developing or improving existing adherence support services that are offered by a multidisciplinary team (nurse, physician, pharmacy, patient et cetera) [6]; directly observed therapy (involving a health care worker, community care worker or family member directly monitoring patients as they swallow their TB medication) [4]; staff motivation and supervision [4]; education and counseling [7]; reminder systems and late patient appointment tracers to help patients keep appointments [8]; incentives and enablers [9]; contracts or written or verbal agreements to return for an appointment or course of treatment; social support provided by community health care workers [10]; social support offered to family members to assist the patient in being adherent, and social support provided by other patients and support groups [6]. These interventions or complex combinations of the interventions may need to be employed to promote TB medication adherence.

Mobile phone text messaging, using the short messaging service (SMS), has recently been proposed as a means of promoting TB medication adherence. The findings may be applicable to adherence to treatment regimens for other conditions such as HIV/AIDS [11], diabetes, asthma as well as cardiovascular disease [12,13]. For promoting adherence to TB treatment, text messages can be sent weekly or daily to patients to remind them to take their medication [11,14] through one-way communication or two-way interactive communication (that is, patients can receive and reply to messages ) [12,15,16]. Text messages may also be used to notify health care providers that the patient has taken their medication [14,17,18]. In addition, the text message intervention can be delivered alone or bundled with economic incentives [14,18]. Globally, mobile phone use is rapidly increasing, with an estimated six billion mobile phone users worldwide at the end of 2011 [5,6]. In particular, mobile phone text messaging has gained popularity among people living in low- and middle-income countries [19]. We therefore propose to conduct a review of the current best evidence for the use of mobile phone text messaging to promote patient adherence to TB treatment.

## Methods

### Criteria for considering studies for this review

#### Type of studies

We will include randomized controlled trials (RCTs). However, non-randomized studies will be considered if there is an inadequate number of RCTs.

#### Types of participants

Participants will be adults (including pregnant women) or children receiving treatment for TB infection, in any setting.

#### Types of interventions

We will include interventions in which mobile phone text messages are used to promote adherence to TB treatment. The text messaging needs to be delivered to a patient with TB, or in the case of an infant or child, to a caregiver. We also will include studies in which the intervention is compared to no intervention, or to other interventions for promoting adherence. We will exclude: studies in which mobile phone voice speaking or voice messaging are interventions; studies in which the use of a beeper or pager is the intervention; studies in which the use of multimedia messaging service is the intervention, and studies in which text messages are bundled with other interventions, unless it is possible to separate the effects of text messaging alone.

#### Types of outcome measures

##### Primary outcomes

The primary outcomes are as follows: treatment adherence; TB cure; successful completion of TB treatment, and development of drug resistance.

##### Secondary outcomes

The secondary outcomes are: exposure to stigma associated with TB as a result of the SMS revealing the patients disease status, and patient satisfaction with the SMS intervention.

### Search methods for identification of studies

A comprehensive and exhaustive search will be performed by MN with the help of the University librarian, to identify all relevant studies available by 30 November 2012, regardless of language or publication status (published, unpublished, in press or in progress). We will search both peer-reviewed journal articles and the grey literature (non-published, internal or non-reviewed papers, reports).

### Database

We will search the following electronic databases: PubMed; EMBASE; Cochrane Central Register of Controlled Trials (CENTRAL); ISI Web of Science (Science Citation Index); Africa-Wide Information, Cumulative Index of Nursing and Allied Health (CINAHL), and WHO library databases (WHOLIS). We will use both text words and medical subject heading (MeSH) terms; for example tuberculosis, patient compliance, mobile phone, text messaging, text*, reminder systems, telemedicine, mHealth, eHealth, medication adherence, and medication compliance. These terms will be used in varying combinations. The literature search strategy will be adapted to suit each database. Table 1 shows the main search strategy we will use.

**Table 1:** PubMed search strategy, modified as appropriate for use in the other databases


**Search**	**PubMed**
#1	“tuberculosis”[MeSH] OR “tuberculosis”[tiab]
#2	“cellular phone”[MeSH] OR “reminder systems”[MeSH] OR telemedicine[MeSH] OR “wireless technology”[MeSH] OR “text messaging”[MeSH] OR text*[MeSH] OR “medical informatics applications”[MeSH] OR SMS[tiab] OR MMS[tiab] OR “mobile phone”[tiab] OR mHealth[tiab] OR “mobile health”[tiab]
#3	“medication adherence” [MeSH] OR “patient compliance”[MeSH] OR adherence [tiab] OR compliance [tiab]
#4	#1 AND #2 AND #3

MeSH, medical subject heading.

### Conference proceedings

We will search the following conference proceedings for relevant abstracts: The Union World Conference on Lung Health, Conference of the Union Africa Region, Conference of the Union Europe Region, Conference of the Union Latin America Region, Conference of the Union Middle East Region, Conference of the Union North America Region, Conference of the Union South-East Asia Region, Conference of the Union Asia Pacific Region, South African Tuberculosis Conference, National Conference on Tuberculosis and Chest Disease (NATCON).

### Searching other sources

In the case of unpublished or ongoing studies, we will search the WHO International Clinical trials Registry Platform, Clinicaltrials.gov, Pan African Clinical Trials Registry (PACTR), and contact individual researchers working in the field as well as the following organizations: WHO, The International Union Against Tuberculosis and Lung disease (The Union), Centers for Disease Control and Prevention and mHealth Alliance. We will also search the website of mHealth Alliance and mHealth in the Low Resource Settings resources database [20] for eligible studies.

### Reference lists

We will obtain reference lists of relevant studies identified and the full-text articles reviewed for inclusion in the review will be checked for additional information.

### Data collection and analysis

The methodology for data collection and analysis will be based on the guidance of the Cochrane Handbook of Systematic Reviews for Interventions [21].

### Selection of studies

We will develop and pilot a screening guide to ensure that the inclusion criteria are adhered to and consistently applied by all review authors. Two review authors (MN and CW), working independently, will screen the titles and abstracts of all studies identified through the literature searches for eligibility. MN will obtain the full text of studies deemed potentially eligible. The two authors (MN and CW) will independently assess the full text of each article for eligibity, and compare their results and resolve discrepancies by discussion and consensus, consulting a third author (LGB, RW or GH) to resolve any persistent disagreements. For all studies excluded by the assessors we will describe the reasons for exclusion.

### Data extraction and management

References will be managed using Thomson ISI ResearchSoft Endnote 9.0 [22]. Two authors will independently extract descriptive and outcome data for each included article using a standardized data collection form, resolving any discrepancies by discussion and consensus; failing which, a third author (LGB, GH or RW) will arbitrate. MN will enter the final data into the Cochrane Collaboration Review Manager version 5.1 statistical software ( http://ims.cochrane.org/RevMan). CW will cross-check the data entered to ensure that there are no data entry errors.

### Assessment of risk of bias in included studies

Two authors will independently assess the risk of bias in the included studies. Separate criteria will be used to assess RCTs and non-randomized studies. The criteria used to assess the risk of bias of in RCTs will be random sequence generation; allocation concealment; blinding of participants, study personnel; blinding of outcome assessors; incomplete outcome data; selective outcome reporting; other sources of bias, and overall risk of bias, in accordance with the methods used by the Cochrane Collaboration [21]. The criteria used for risk of bias assessment for non-randomized studies will include selection bias (with regard to comparability of groups, confounding and adjustment); performance bias (in terms of the fidelity of the interventions, and quality of the information regarding who received which interventions, including blinding of study subjects and healthcare providers); detection bias (regarding unbiased and correct assessment of outcomes, including blinding of assessors); attrition bias (with regard to completeness of sample, follow-up and data), and reporting bias (with regard to publication biases and selective reporting of results) [21]. Studies will be scored as having low, high or unclear risk of bias. The two authors will resolve disagreements in the assessment of risk of bias by discussion and consensus, consulting a third author to resolve any persistent disagreements.

### Measures of treatment effect

Data analysis will be conducted using the Cochrane Collaboration Review Manager version 5.1 statistical software ( http://ims.cochrane.org/RevMan). The outcomes of interest will be either dichotomous or continuous. We will calculate risk ratios and their corresponding 95% confidence intervals and *P*-values for dichotomous outcomes, and mean differences for continuous outcomes.

### Dealing with missing data

In cases of missing or incomplete information presented in the included studies, we shall contact authors for further information.

### Data synthesis, assessment/investigation of heterogeneity

We will assess clinical heterogeneity by examining types of participants, interventions and outcomes in each study. We will pool data only from studies judged to be clinically homogenous. Statistical heterogeneity in each meta-analysis will be assessed using the chi-square test and quantified using the *I*-squared statistic. If studies are sufficiently homogenous (in terms of study populations, interventions and outcomes), then we will pool the data across studies and estimate summary effect sizes using a fixed-effect model. Otherwise, we will use the random-effects model. We will perform subgroup analyses by intervention subtypes: long versus short messages; daily versus weekly messages; short weekly messages versus long weekly messages; short daily messages versus long daily messages, and two-way interactive communication versus one-way communication [11,12,15,16]. We will also stratify analysis by study design (RCTs separate from non-randomized studies). Finally, we will use the grading of recommendations assessment, development, and evaluation (GRADE) approach [23] to assess the quality of evidence for the effectiveness of the SMS intervention. This method results in an assessment of the quality of the body of evidence as high, moderate, low, or very low. Evidence is considered of high quality if ‘further research is very unlikely to change our confidence in the estimate of effect; and moderate quality if ‘further research is likely to have an important impact on our confidence in the estimate of effect and may change the estimate. Low quality evidence implies that ‘further research is very likely to have an important impact on our confidence in the estimate of effect and is likely to change the estimate, and very low quality that ‘we have very little confidence in the effect estimate.

### Sensitivity analyses

Several sensitivity analyses will be performed: first to determine whether the study design (RCT versus non-randomized study) could influence the results of the meta-analysis; second, to evaluate whether the model of the statistical method (random-effect vs fixed-effect model) could change the results, and third, to determine the impact of excluding studies with a high risk bias on the results, with emphasis on allocation concealment, blinded outcome assessment, and losses to follow-up (with a cut off of 25% loss to follow-up).

### Presenting and reporting of results

Findings in our systematic review will be presented in several ways. Flow diagrams will be used to summarise the study selection process. Funnel plots will be used to assess publication bias if we identify 10 or more eligible studies. The kappa statistic [24] will be used to assess agreements between the full-text screening, data extraction and risk of bias assessment by the two authors (MN and CW). GRADE summary of tables of findings, risk of bias tables or graphs, and forest plots will also be used where appropriate. The reporting of outcomes without quantitative data will be descriptive. Lastly, we will provide a list of excluded studies with reasons for exclusion

## Discussion

### Expected significance of the study

The findings of this systematic review will have implications for policy, practice and research. Our results will provide evidence of whether or not policy makers can adopt SMS adherence intervention as best practice to be used alone or in combination with other proven adherence interventions such as DOTs. They will also inform clinic or hospital managers of how best to use the intervention to promote adherence thereby achieving high cure rates and low treatment-failure rates among patients while decreasing the patient load for DOTs staff [25]. The systematic review may also identify specific considerations that would need to be taken into account for future studies, such as study location; content and timing of messages; whether or not patients replied to text messages; how text messages where sent (automated versus manual); measurement of adherence; variety of text messages sent (inclusion of jokes or lifestyle tips); duration of the study; whether or not participants were provided with the mobile handsets, and and sample size [13].

## Abbreviations

CENTRAL: Cochrane Central Register of Controlled Trials; CINAHL: Cumulative Index of Nursing and Allied Health; DOTS: directly observed treatment short course; GRADE: grading of recommendations assessment development, and evaluation; MeSH: medical subject heading; NATCON: National Conference on Tuberculosis and Chest Disease; PACTR: Pan African Clinical Trials Registry; RCT: randomized controlled trial; SMS: short messaging service; TB: Tuberculosis; WHO: World Health Organization; WHOLIS: World Health Organization (WHO) library databases.

## Competing interest

The authors declare that that they have no competing interests.

## Authors’ contributions

MN and CW contributed to the conception and design of the review, and will be involved in data acquisition. MN will analyze the data with input from all co-authors, and all authors will participate in the interpretation of the results. All authors were involved in the drafting of this protocol and have given their approval for publication.
